# A Novel Method Incorporating Gene Ontology Information for Unsupervised Clustering and Feature Selection

**DOI:** 10.1371/journal.pone.0003860

**Published:** 2008-12-04

**Authors:** Shireesh Srivastava, Linxia Zhang, Rong Jin, Christina Chan

**Affiliations:** 1 Department of Chemical Engineering and Materials Science, Michigan State University, East Lansing, Michigan, United States of America; 2 Department of Computer Science and Engineering, Michigan State University, East Lansing, Michigan, United States of America; 3 Department of Biochemistry and Molecular Biology, Michigan State University, East Lansing, Michigan, United States of America; University of Michigan, United States of America

## Abstract

**Background:**

Among the primary goals of microarray analysis is the identification of genes that could distinguish between different phenotypes (feature selection). Previous studies indicate that incorporating prior information of the genes' function could help identify physiologically relevant features. However, current methods that incorporate prior functional information do not provide a relative estimate of the effect of different genes on the biological processes of interest.

**Results:**

Here, we present a method that integrates gene ontology (GO) information and expression data using Bayesian regression mixture models to perform *unsupervised clustering* of the samples and identify physiologically relevant discriminating features. As a model application, the method was applied to identify the genes that play a role in the cytotoxic responses of human hepatoblastoma cell line (HepG2) to saturated fatty acid (SFA) and tumor necrosis factor (TNF)-α, as compared to the non-toxic response to the unsaturated FFAs (UFA) and TNF-α. Incorporation of prior knowledge led to a better discrimination of the toxic phenotypes from the others. The model identified roles of lysosomal ATPases and adenylate cyclase (AC9) in the toxicity of palmitate. To validate the role of AC in palmitate-treated cells, we measured the intracellular levels of cyclic AMP (cAMP). The cAMP levels were found to be significantly reduced by palmitate treatment and not by the other FFAs, in accordance with the model selection of AC9.

**Conclusions:**

A framework is presented that incorporates prior ontology information, which helped to (a) perform unsupervised clustering of the phenotypes, and (b) identify the genes relevant to each cluster of phenotypes. We demonstrate the proposed framework by applying it to identify physiologically-relevant feature genes that conferred differential toxicity to saturated vs. unsaturated FFAs. The framework can be applied to other problems to efficiently integrate ontology information and expression data in order to identify feature genes.

## Introduction

Current methods of feature selection can be classified into two major categories: data-based and prior information-based. The data-based techniques rely primarily on the microarray data and sophisticated modeling or machine trained under the conditions of supervised classification to identify the distinguishing features (genes). The simpler ‘filtering’ techniques classify the subgroups by maximizing the ratio of between-group to within-group variance. Examples of filtering techniques include the Wilcoxon's rank sum test [1], Fisher's Discriminant Analysis (FDA) [2], [3], discriminative partial least squares (PLS) [4] or genetic algorithm (GA)- [5] based classification and clustering [6], [7]. However, these techniques suffer from certain drawbacks, e.g., many among them are based on methods that require the genes to be independent and uncorrelated, which microarray data is not [8]. Therefore, improvements to the filtering techniques have been made, such as “minimum redundancy and maximum relevance (mRMR)” [8]. Additionally, sophisticated ‘wrapper’ techniques have been developed, which employ a trained learning machine to identify the relevance of genes to a phenotype. Examples of wrapper techniques include support vector machines (SVM) [9] and the generalized least absolute shrinkage and selection operator (LASSO) [10], [11]. The wrapper methods are considered better than the filter methods because they can incorporate the inter-correlation of genes and can also determine the optimal number of variables. A third set of techniques are also being developed which combine the wrapper and the filter techniques (e.g. the kernel Fisher discriminant analysis, KFDA) [12] or multi-layer perceptrons [13]. There are two major shortcomings with the existing feature selection approaches. First, these approaches do not incorporate the vast amount of information already available on the functions of the genes. Typically, the functional information of the genes is employed only in the post-processing of the selected genes. The incorporation of prior knowledge of genes is particularly important when the expression data is noisy. Second, most of the feature selection approaches belong to a family of supervised discriminative analysis and therefore require labeling information of the phenotypes (i.e., which phenotypes are in the same group) to identify feature genes.

In order to address the first issue, alternative analysis methods are being developed which incorporate prior information of the genes [14]–[16]. In these knowledge-based methods, the association of a pre-defined gene ontology (GO) category to a phenotype is statistically evaluated, and used to identify the discriminating cellular processes [17]–[19]. Such GO-based techniques identify the gene sets and subsets which have significant association to a phenotype. Individual genes which may be significantly altered and have important association with the phenotype may not be selected if the gene-group they belong to is not enriched. Even among the subsets that are identified as important, the identification of a few (one or two) targets can be difficult and subjective. Another major drawback of these approaches is the tedious manual preparation and updating of the data sets. While data sets are available for Affymetrix chip for some species, for other platforms, such as the custom cDNA microarrays, one would need to manually define the gene sets, which can delay procurement of downstream information or introduce errors. Therefore, such techniques are useful when the expressions of many genes of an important, causative pathway change. For other situations where there is change of only a few, rate-controlling genes, such approaches may not be as informative. Nevertheless, applications of these techniques have lent support to the notion that incorporation of prior knowledge could either improve the classification efficiency or identify more relevant biological processes. Regarding the second issue, a few recent studies have aimed to combine unsupervised data clustering with feature selection. In [20], the authors proposed altering the procedure of data clustering and feature selection iteratively. In each iteration, the data points are first clustered according to the selected features, and then FDA is applied to identify a new set of features according to the cluster labels. In [21], [22], the iterative procedure is improved by converting the original problem into a convex optimization problem. However, none of these studies are able to exploit the prior knowledge of the data, which is important with microarray data analysis.

Here, we present a general framework for feature selection that is able to overcome the two shortcomings simultaneously. The proposed framework integrates the ontology information of the genes with their expression data (X) to (a) perform unsupervised data clustering to group similar cellular responses (Y) into clusters, and (b) to identify the genes that are most discriminative among the clusters of cellular responses. Mixture regression models are first applied to cluster the multiple experimental conditions. Important genes with high correlation to each group of experimental conditions are then found by a regression model that automatically incorporates the GO information. The key genes that differentiate the groups of conditions are identified to provide insight into the differences among the biological processes. A major advantage of this method is the easy assimilation and update of the functional information of the genes. Another major advantage of the proposed method is that it unifies unsupervised data clustering with supervised feature selection into a single framework. This combination allows us to identify genes relevant to multiple biological processes without having to know, *a priori*, which experimental condition is related to which biological process. This is important when conditions are difficult to classify or the classification of conditions are unknown *a priori*.

Finally, the proposed method allows for parallel identification of genes relevant to multiple cellular responses, which makes it an efficient high-throughput analysis.

We demonstrated the proposed method by applying it to identify the genes that are likely involved in the toxicity of FFAs, in particular saturated (SFA), palmitate, and TNF-α. Our experimental results showed that our proposed method is able to (a) identify the group of toxic experimental conditions, and (b) identify the genes that are relevant to the toxic conditions.

## Methods

### Bayesian Regression Model

The central assumption behind this method is that the genes within a GO category would have similar effect on a cellular response. Therefore, genes belonging to the same GO category were constrained to have similar regression weights. The above assumption may not always hold, by employing a restrictive assumption, we aim to significantly reduce the hypothesis space of the regression model. We believe that given a large number of genes and a small number of experimental conditions, it is more important to restrict the hypothesis space for data fitting. This decision is also supported by our experimental results (below). Additionally, in order to circumvent the noise typically associated with microarray data, the gene expressions were approximated by a multivariate Gaussian distribution and the regression weights were estimated by minimizing the regression error that was averaged over the Gaussian distribution.

Let *X* = (**x**_1_, **x**_2_,…, **x** *_m_*) denote the gene expression data for *m* different experimental conditions, where **x** *_k_* = (*x*_1_, *x*_2_,…, *x_n_*) represents the expression data of *n* genes under the *k*th condition. Let **y** = (*y*_1_, *y*_2_,…, *y_m_*) denote the corresponding cellular responses for the *m* conditions. By assuming that the conditional probability Pr(**y**|*X*, **w**) follows the Gaussian distribution *N*(*X^T^* **w**, *σ* ^2^ *I*), the regression error could be computed as
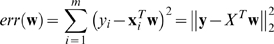
(1)where, **w** = (*w*_1_, *w*_2_,…, *w_n_*) are the regression weight assigned to the *n* genes. The optimal solution for **w** that minimizes the above regression error is
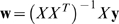
(2)Now, consider multiple replicates of the gene expression data under each experimental condition. Let 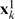, 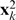,…, 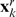 denote the *r* replicates of the gene expression data under the *k*th condition. We can approximate the distribution of gene expression data under the *k*th condition, i.e., Pr(**x** *_k_*), by a Gaussian distribution *N*(**x̅** *_k_*, *S_k_*) where **x̅** *_k_* and *S_k_* are calculated as follows:
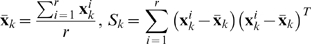
(3)Due to the limited number of replicates (i.e., *r* is small for most conditions), we simplify *S_k_* as a diagonal matrix by setting the off-diagonal elements of *S_k_* to be zero. This is particularly important for our study since there are thousands of genes involved in the process, and the number of replicates is only two, which makes it impossible to estimate any off diagonal elements in *S_k_*. Using **x̅** *_k_* and *S_k_* in (3), instead of regressing the cellular response to the averaged gene expression data **x̅** *_k_*, the Bayesian regression model will search for the regression weights **w** that minimize the following *expected* regression error:
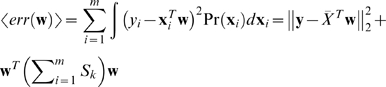
(4)where, *X̅* = (**x̅**_1_, **x̅**_2_,…, **x̅** *_m_*). The optimal solution for the Bayesian regression model is
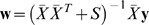
(5)where, 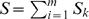. Comparing the above expression for **w** to the expression in (2), we see that the primary difference between the two expressions is that (5) incorporates covariance matrix *S* into its denominator. The introduction of S will assign smaller regression weights to the genes whose expression data exhibit large variance compared to the genes with small variance. Finally, genes with the largest absolute regression weights are deemed to be the most important genes and are selected for analysis. Note that equation (5) is essentially similar to the ridge regression model. As suggested by several studies (Roth, 2004), Lasso regression using L1 norm tends to achieve better performance for feature selection. However, the ridge regression is computationally more efficient compared to Lasso regression. This is particularly important since our approach employs an iterative algorithm to approximate the optimal regression weights, and therefore efficient computation is essential to our approach.

### Mixture Model

The main idea behind the mixture models is to cluster the experimental conditions into an optimal number of subgroups and build a different regression model that relates the gene expression data (X) to a cell response (Y) for each subgroup. The clustering of experimental conditions, however, is based on their regression weights. For example, two experimental conditions will be grouped into the same cluster if they share similar regression weights. However, the regression weights of each experimental condition would also depend on the clustering results because a regression model can be built only for a group of experimental conditions. Hence, the technical challenge of regression mixture model lies in resolving this dilemma. We applied Expectation Maximization (EM) algorithm to effectively resolve this problem. The key idea behind the EM algorithm is to iteratively alternate the clustering and the regression procedures. At the very beginning of the EM algorithm, experimental conditions are randomly assigned to clusters and a regression model is built for each cluster. Then, the regression weights obtained for the genes are used to regroup the experimental conditions into a new set of clusters, and the new clustering results are used to generate new regression weights for the genes. The clustering and the regression procedures alternate until a stable solution is reached where the parameters no longer change with further iterations. It can be shown that the EM algorithm described above will indeed maximize the log-likelihood of the gene expression data. Furthermore, the iterative procedure is guaranteed to converge to a solution that is a local maximum [23].

In the regression mixture model, we don't assume that all the experimental conditions share the same regression weights **w**. Instead, we assume that there are *K* (*K*<*m*) different sets of regression weights, one for each sub-population. Each experimental condition will choose the most suitable set of regression weights. Below, we outline the key idea behind the variational EM algorithm that is used in our calculation.

We model the conditional probability Pr(**y**|*X*, **w**) by
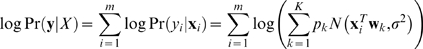
(6)In order to incorporate the variance in the gene expression data, similar to the Bayesian regression model, we compute the expected log Pr(**y**|*X*) that is averaged over the distribution of the gene expression data *X*. More specifically, the expected log Pr(**y**|*X*) is computed as
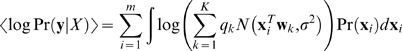
(7)Where *q_k_* is the prior for choosing the *k*th regression model. To facilitate the computation, we follow the idea of variational method by introducing a variational distribution *φ_i_*_,*k*_ = Pr(*k*|*y_i_*, **x** *_i_*), *i* = 1,…,*m*, *k* = 1,…, *K* and approximate the log-likelihood expression in (7) as follows:
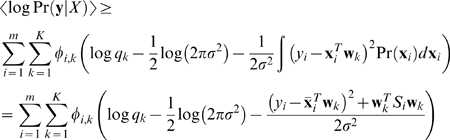
(8)Thus, we have the following updating equations to compute *φ_i_*_,*k*_, *q_k_*, **w** *_k_*, and *σ* ^2^

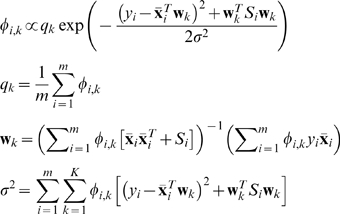
(9)We further improved the robustness of the model by introducing the prior for the regression weights Pr(**w** *_k_*) as a Gaussian distribution *N*(**0**, *λ* ^−1^). Then, instead of maximizing the log-likelihood, we will maximize the logarithm of the posterior probability, i.e.,
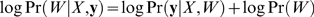
(10)where, *W* = (**w**_1_, **w**_2_,…, **w** *_K_*). The updating equations in (9) are unchanged except that the equation for **w** *_k_* is changed to the following
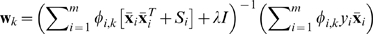
(11)Evidently, the introduction of the uninformative prior is in general to reduce the magnitude of the regression weights. As a result, the small weights will become smaller and even zero, which could result in a sparse solution for *W*.

We summarize the EM algorithm used in our calculation as follows:

For t = 1, 2, …

1. E-step: compute *φ_i_*_,*k*_ in (9) for each condition and every mixture model
2. M-step: compute *q_k_* and σ in (9), and **w** *_k_* in (11)

### Incorporation of GO information into the similarity matrix

To incorporate the GO information into the regression model, we first represent each gene by the set of GO terms that are associated with the gene. We further expand the GO profile of each gene by including the parent nodes of each associated GO code. We then compute the similarity between two genes based on the overlap between their GO profiles. Since some GO codes may be more important than others, we adopt the term frequency independent document frequency (TF.IDF) weighting scheme of information retrieval and weigh each GO code by the IDF factor when computing the gene similarity. The IDF factor for a GO code *g* is computed as
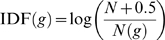
(12)where *N* is the total number of genes and *N*(*g*) is the number of genes whose profile include the GO code *g*. The advantage of using the IDF weight is that it down weighs the common GO codes while computing gene similarity. This is based on the assumption that a GO code is likely to be less important in deciding the similarity between two genes if it is commonly shared by a large number of genes. For example, the GO code ‘mitochondrial genes’ has many genes which belong to mitochondria but may not be functionally related. We denote the pairwise gene similarity by the matrix *T* = [*T_i_*_,*j*_]*_n_*_×*n*_ where element *T_i_*_,*j*_ represents the similarity between the *i*th gene and the *j*th gene. We would like to emphasize that the above assumption may not hold in some biological processes. In particular, two genes sharing a large similarity in their GO functions may show opposite effects on regulating a phenotype. The situation could be even more complicated when one gene up-regulates the expression levels of certain genes under some conditions and down-regulates their expression levels under other conditions.

Based on this assumption, we can construct an energy function to measure the consistency between the assigned regression weights **w** and the gene similarity *T*, as shown below:
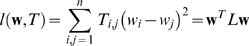
(13)where *L* is the graph Laplacian of similarity matrix *T*. Evidently, the smaller the *l*(**w**, *T*) is, the more consistent the regression weight **w** is to the gene similarity *T*. We can then incorporate the gene similarity *T* into the regression model as a Bayesian prior for regression weights *W*, i.e.,
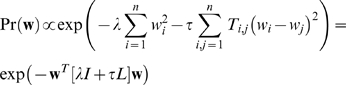
(14)Note that in the above, in addition to the prior for the gene similarity *T*, we also include the uninformative prior through the factor *λI*. The updating equations for the integrated Bayesian regression mixture model are almost identical to the ones in (10) except that the equation for **w** *_k_* is changed to the following:
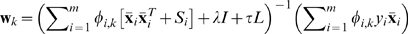
(15)


### User-specified parameters of the Integrative Mixture GO (IMGO) model

The two user-specified parameters to our mixture model are λ and τ. Parameter λ is related to the uninformative prior and its role is to reduce the variance in the regression weights. Given that we have a large number of genes and relatively small number of experimental conditions, there can be an infinite number of ways to regress the cell response that are equally valid. The introduction of the uninformative prior *λI* will allow us to distinguish among the regression models that have the same regression error. In particular, by increasing the value of the parameter λ, we require the regression model to assign large weights to only a small number of genes and most genes are assigned very small or even zero weights. In other words, λ is used to control the volume of the solution space for the regression weights. **A large λ will lead to a smaller solution space** and vice versa. The parameter τ defines the weight that is assigned to the GO information. **The larger the τ, the more we require the regression weights to be consistent with the GO information**.

In addition to λ and τ, we also have to determine the number of clusters when applying the mixture model. In our case, the number of clusters is decided by the application. In particular, we have prior knowledge that the experimental conditions can be classified into toxic vs. non-toxic conditions. Hence, the experimental conditions clustered naturally into two groups, in this application.

### Extension to the Case When the Classification of Experimental Conditions is Given

In the framework proposed above, we assume that the classification of experimental conditions is unknown, which is automatically discovered by the mixture model. In this section, we demonstrate that the proposed framework can be easily extended to scenarios where the classification of experimental conditions is provided. We denote by *κ_i_*_,*k*_ the classification of the experimental conditions: *κ_i_*_,*k*_ = 1 indicates that the *i*-th experimental condition belong to the *k*-th group of conditions, and zero otherwise. We modify equation (15) by replacing *φ_i_*_,*k*_, the group assignment computed by the mixture model, with *κ_i_*_,*k*_, the given classification information for the experimental conditions. The resulting expression for the regression weights is
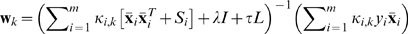
(16)We refer to this method as the “**simpler method**” to differentiate it from the mixture regression model that is proposed above.

## Results

### Application to identify genes associated with fatty acid toxicity to human hepatoma cell line

**Figure 1:**
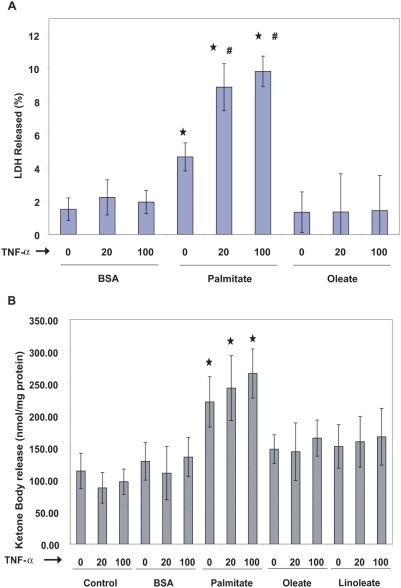
The cytotoxicity and ketone body production in response to various treatments. Confluent HepG2 cells were treated for 24 h with 0.7 mM of the indicated FFA complexed to 4% (w/v) BSA, in the presence or absence of TNF-α (0, 20 or 100 ng/ml). (A) Cytotoxicity of the treatments. The cytotoxicity was measured as the % LDH released, as defined in the methods section. (B) Ketone body production. Acetoacetate and beta-hydroxybutyrate release into the media were measured by enzymatic assays. Ketone body release was calculated as the sum of acetoacetate and beta-hydroxybutyrate release. Data presented as mean±s.d. of three independent experiments. ★, significant FFA effect, p<0.01, #, significant TNF-α effect, p<0.01.

Free fatty acids and TNF-α have been suggested to play important role in lipotoxicity. Yet, it is not clear which genes may play a role in lipotoxicity in hepatocytes. Additionally, it is not clear whether this interaction is affected by the type of free fatty acids. It is known that exposure to elevated FFAs could cause lipotoxicity, i.e., cell death associated with excessive lipid accumulation or exposure. It has been also discovered that saturated FFAs are more cytotoxic at elevated physiological concentrations than the unsaturated FFAs. However, the underlying changes associated with the differential toxicity of saturated and unsaturated FFAs are not clearly known. Additionally, TNF-α is another factor that has been implicated in obesity-associated disorders. We treated human hepatoma cell line, HepG2 cells, with elevated physiological level (0.7 mM) of different types of FFAs (saturated, monounsaturated and polyunsaturated) “crossed” with 3 different levels of TNF-α, 0, 20 and 100 ng/ml for 24 h. Global gene expressions were measured by microarray analyses. LDH release was measured as a marker of lipotoxicity. It was observed that saturated FFA was much more toxic than unsaturated FFAs and TNF exposure further increased the toxicity of the saturated FFA (Figure 1A). Cells exposed to saturated FFAs also had significantly higher ketone body release as compared to cells treated with unsaturated FFAs (Figure 1B). We applied IMGO model to automatically identify the cytotoxic and ketogenic conditions and the underlying gene changes that may be associated with the differential cytotoxic response of liver cells to the different types of FFAs and TNF-α.

We first conduct experiments using the simpler method by assuming the classification of the experimental conditions is given. We manually define the cytotoxic conditions and select the genes using the computational model. The top five genes identified by this analysis are shown in Table 1. We found that caspase 6, one of the selected genes has been shown to play a role in the toxicity of saturated FFAs to other cell type but not with our cell type (data not shown). However, for the other four genes identified by the simpler method, they have not been shown to play a direct role in lipotoxicity according to existing literature [24]–[28]. Overall, the results with the simpler method are mixed.

**Table 1:** Top 5 genes identified by supervised clustering-based model (“simpler method”).

Parameter Values	LL id	Name
λ = 0.5	3486	(gC) insulin-like growth factor binding protein 3 (IGFBP3), mRNA. (AA598601,NM_000598,Hs.77326)
τ = 3	3632	(gN) inositol polyphosphate-5-phosphatase, 40 kDa (INPP5A), mRNA. (T58773,NM_005539,Hs.124029)
	10537	(gC) ubiquitin D (UBD), mRNA. (N33920,NM_006398,Hs.44532)
	839	(gN) caspase 6, apoptosis-related cysteine protease (CASP6), transcript variant beta, mRNA. (W45688,NM_032992,Hs.3280)
	51704	(gC) G protein-coupled receptor, family C, group 5, member B (GPRC5B), mRNA. (W35153,NM_016235,Hs.242407)

**Figure 2:**
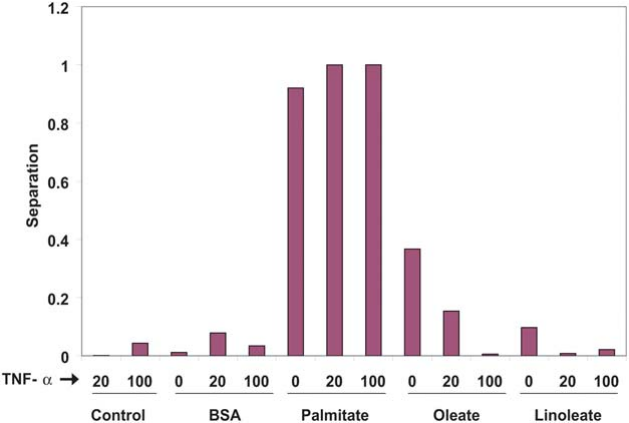
Discrimination of cytotoxic conditions by the IMGO analysis. The ability of the two-population model for cytotoxicity to distinguish the cytotoxic (high LDH release) conditions was tested.

In the second experiment, we examined the proposed framework without *a priori* knowledge of the classification of the experimental conditions. First, we showed that the proposed framework is able to identify the group of cytotoxic and ketogenic conditions and the group of non-toxic conditions. This is illustrated in Figure 2, in which a “separation” score, computed based on the difference between the probabilities of assigning to the two groups, is plotted for each experimental condition. We clearly see that for all the cytotoxic and ketogenic conditions, their separation scores are much larger than that of the non-toxic conditions, indicating a clear separation between the two groups of experimental conditions. This result indicates that the unsupervised clustering method proposed in this paper is able to automatically identify the experimental conditions that are in the same group. The parameter values used for this analysis were: lambda = 0.5 and tau = 3. The details of the selection of the parameter values are provided in the supplementary file S1 and supplementary figure S1.

**Figure 3:**
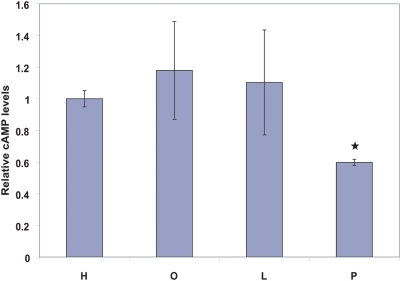
Effect of FFA-treatments on intracellular cAMP levels. Cells were treated for 24 h with 0.7 mM of different types of FFA and the levels of intracellular cAMP were measured. H = Control medium, O = 0.7 mM oleate, L = 0.7 mM linoleate, and P = 0.7 mM palmitate. ★, p<0.01 by a two-tailed t-test.

Second, we examine the genes identified by the proposed framework that are listed in Table 2. Lysosomal ATPases were selected as the top genes. This identified an important role of lysosomes in the toxicity of palmitate to the HepG2 cells. A previous study [29] has shown that the cytotoxicity by palmitate to hepatocytes could be reduced by reducing lysosomal permeabilization. Another important gene identified by the analysis is adenylate cyclase 9 (AC9). The selection of AC9 only for the cases with high separation suggested that cAMP levels should be differentially modulated by palmitate treatment and not by unsaturated FFAs. This was indeed found to be the case (Figure 3). Thus, the model was able to identify the genes that are altered by saturated FFAs and play a role in the toxicity.

**Table 2:** Top 5 identified genes by IMGO for various values of separation.

Parameter Values	Separation	LL id	Names
λ = 0.5	0.9208	535	(gN) ATPase, H+ transporting, lysosomal V0 subunit a isoform 1 (ATP6V0A1), mRNA. (AA430654,NM_005177,Hs.267871)
τ = 3		115	(gF) adenylate cyclase 9_(H64281,_,Hs.20196)
		4792	(g) nuclear factor of kappa light polypeptide gene enhancer in B-cells inhibitor, alpha (NFKBIA), mRNA. (W56300,NM_020529,Hs.81328)
		9550	(gC) ATPase, H+ transporting, lysosomal 13 kDa, V1 subunit G isoform 1 (ATP6V1G1), mRNA. (AA608567,NM_004888,Hs.90336)
		1183	(gC) chloride channel 4_(AA019316,_,Hs.199250)

Mixture model with two sub-populations were fitted to the data for the values of the parameters λ and τ shown, and the difference in the probabilities of the two models that fit the P-0 condition was calculated. For the choice of the parameters shown, the two models had very different probabilities that fit the palmitate condition, whereby one model had a much greater probability than the other in fitting the palmitate results. For such scenario, genes with the greatest difference of weights for the two populations are shown as they represent genes that have the greatest differential effect on the toxicity, or are responsible for differentiating the toxic condition.

## Discussion

Incorporation of functional information of the genes in the microarray analysis is an active area of research. However, most of the currently available methods utilize the prior functional (GO) information to generate pre-defined sets of genes [17], [18]. The underlying assumption in these analyses is that a phenotype is altered by concerted changes in the expression of many genes of a GO-category [17]. Though the approach presented here is conceptually similar to these approaches, there are some major differences. (a) Unlike most existing studies that are based on supervised feature selection, our study applies unsupervised feature selection. Specifically, we unified unsupervised data clustering with supervised feature selection under the same framework, which allows us to identify the feature genes even though the classification of the experimental conditions is unknown. (b) The similarity matrix employed in the analysis is based on the overlap in the GO profiles among every pair of genes in the dataset. Therefore, there are no strict gene sets and the possibility of interaction/ coregulation of any pair of genes is incorporated as well as weighted, i.e., genes with greater overlap in their GO-profile have a greater coefficient in the similarity matrix and vice versa. (c) One can control the contribution of the GO information in the model. In our study, the contribution of the similarity matrix (GO information) is weighted by the factor τ, which can be varied. This is in contrast to other methods where the GO information takes precedence over the subsequent analysis. One disadvantage of methods based centrally on GO information is that the prior knowledge is typically generated for certain sets of conditions and cell-types, so that the information generated may not be universally valid (for every cell type and treatment condition). Our experience has shown that there exists an optimal value of the GO-contribution (τ), beyond which the separation deteriorates. This method allows one to take this into consideration and control the GO-contribution to achieve the best discrimination among the subpopulations.

As a representative application, we applied the model to identify the genes associated with toxicity of saturated FFAs to human hepatoma cells. We compared two alternatives for the proposed framework- automatic (unsupervised) clustering and gene selection, and user-defined (supervised) classification and gene selection. The latter is denoted above as the simpler method. As expected, the genes identified by the two methods are different, even though the equations identifying the genes (equations 15 and 16) are fairly similar. The key different between the unsupervised method and the supervised one is that the unsupervised method automatically computes probability *φ_i_*_,*k*_ in (15), which weights the i-th experimental condition for the k-th group. As indicated in Figure 2, this probability varies significantly across the experimental conditions that are in the same group. On the other hand, for the supervised method, the *a priori* classification information of conditions is encoded by a binary variable *κ_i_*_,*k*_, which gives the same weight for all the experimental conditions that are in the same group. We believe that the ability to weigh experimental conditions in the same group differently leads to better gene selection with the unsupervised method.

In conclusion, IMGO is a novel method to integrate prior information and gene expression to identify feature genes which play important role in a cellular phenotype/response as well as those that are affected differently under different conditions.

## Supporting Information

Supplementary File S1 Supplementary file
(0.04 MB DOC)

Figure S1 (6.65 MB EPS)
