# Supplementary material for: A Novel Method Incorporating Gene Ontology Information for Unsupervised Clustering and Feature Selection
Source: PLoS One. 2008 Dec 4;3(12):e3860. doi: 10.1371/journal.pone.0003860 (PMC2585795; doi:10.1371/journal.pone.0003860)
Supplement: Supplementary File S1 — Supplementary file (0.04 MB DOC) [file pone.0003860.s001.doc]

**Supplementary File**

**1.** **Integrative Mixture Model Gene Ontology (IMGO) analysis**

The genes which were affected by the treatments (FFA or TNF-α or their interaction) were identified by two-way ANOVA analysis. In order to identify the genes which contribute to the cytotoxicity of palmitate and TNF-α from the other treatments, the IMGO analysis was performed on the differentially expressed genes. To achieve discrimination of toxic and ketogenic conditions from the others, a two-population mixture model was employed.

**1.1 *Separation* of toxic vs. non-toxic conditions**

The ability of the IMGO to discriminate between the different phenotypes (e.g., cytotoxic vs. non-toxic conditions or high ketogenic vs. low ketogenic conditions) was measured as the difference in the posterior probabilities (i.e., $\varphi_{i,k}$) of assigning P-0 condition (i.e., the palmitate without TNF-α (P-0) condition) to each of the two mixture models. This condition (P-0) was chosen because it defined a threshold value for the toxic phenotype and showed the maximum variation with the parameters (λ and τ). If P-0 condition was separated, the other toxic conditions were separated as well. The separation ranged from 0 to 1, with 0 indicating no separation and 1 indicating perfect separation. For almost 0 separation, the weights assigned to different genes were very similar for the two models. This suggested that essentially a single model would fit the entire data. For some sets of λ and τ a large separation was achieved. This suggests that one of the two models fit the toxic condition much better than the other, while the other model essentially modeled the non-toxic conditions. In this case, the difference in the regression coefficients of a gene for the two models indicated a differential effect on the toxic vs. non-toxic conditions. Such genes, in other words, are those which change differently between the toxic vs. the non-toxic conditions and potentially contributed to the toxicity.

**1.2 Choice of values for the parameters λ and τ**

Since the model contained two parameters λ and τ (see the ‘Model description’ section), the effect of these parameters on the ability of the mixture model to separate the cytotoxic and non-toxic conditions were studied to: (i) identify the optimal values of the two parameters to achieve maximal separation and (ii) evaluate the effect of errors in the S matrix on the separation profile. Figure S1a shows the variation of separation with the two parameters. The separation displayed three peaks at λ = 0.1 and τ = 10 (separation = 0.9917), λ = 0.5 and τ = 3 (separation = 0.9260) and λ = 1 and τ = 1 (separation = 0.9601). For very small λ (= 0.1), the separation increased with τ. This suggests that for large solution space defined by small λ, increased incorporation of prior knowledge provides the necessary additional constraint to improve the classification. For large λ, the separation displayed a maxima at τ = 3. This suggested that for smaller solution space, the incorporation of prior knowledge (τ) improves the discriminating ability of the IMGO, but as the incorporation of prior information is increased, the discrimination between the toxic vs. non-toxic conditions reduces. This is most likely due to the constraint imposed by the higher τ on the weights. A higher τ means that more genes with similar ontology are assigned similar weights, possibly overriding the expression data of the genes, leading to reduced separation. In other words, the solution may be over-constrained. In order to select the most appropriate values of these parameters to identify the important genes, we evaluated the:

1. Effect of noise/ error in the similarity matrix on the separation profile.
2. Effect of deleting conditions on the separation profile.

**1.2.1 Effect of noise/ error in the similarity matrix on the separation profile**

The elements of the similarity matrix were randomly varied by 20% and the effect on the separation profile was observed (Figure S1 b) to be very similar to the base case (Figure S1 a). This indicated that the separation was robust to errors in the similarity matrix.

**1.2.2 Effect of deleting conditions on the separation profile**

In order to test the effects of reduced number of conditions (simulating an incomplete experimental design) on the separation profile, we evaluated the separation profile with a reduced number of conditions. Because the aim was to identify how the separation of toxic conditions is affected by reducing the number of conditions, we tested the effect of removing certain fatty acid and non-fatty acid conditions on the separation of toxic conditions. Removing (H-20 and H-100) or (L-0 and L-100) conditions altered the separation profiles (Figures S1 c and S1 d). In particular it was observed that:

a. Removing these conditions significantly reduced the maximum separation.

b. The peaks at the extremes of λ and τ vanished, while the central peak at λ = 0.5 and τ = 3 remained, although the profile became flatter around the peak for the latter case.

c. Removing the fatty acid (L-0 and L-100) conditions resulted in slightly better separation (max separation = 0.5206) than removing the ones without fatty acid treatments (H-20 and H-100, max separation = 0.4610), although the shapes of the resultant profiles were similar.

While these results emphasized the importance of a more complete experimental design to span the whole experimental area in order to achieve optimal separation, they also suggest that having a less extensive experimental area or conditions may be ameliorated with literature or domain knowledge, e.g. GO information. Given the combination of incomplete experimental area with some domain knowledge, the peak at λ = 0.5 and τ = 3 was fairly robust to noise and removal of experimental conditions, while the peaks at the extremes (representing λ = 0.1 and τ = 10, and λ = 1 and τ = 1) were not. Because of these reasons, the parameter values of λ = 0.5 and τ = 3 were utilized for the subsequent analyses.

**2. Materials and Methods**

**2.1 Materials**

HepG2/C3A cells and Fetal Bovine Serum (FBS) were from American Type Culture Collection (Manassas, VA). Dulbecco’s modified Eagle’s medium (DMEM) contained high glucose but no pyruvate and was obtained from Invitrogen (Carlsbad, CA), Penicillin-Streptomycin (P/S), phosphate buffered saline (PBS, pH 7.4) and Trizol reagent were also from Invitrogen. Fatty acid free bovine serum albumin (BSA) was purchased from MP Biomedicals (Chillicothe, OH). Free fatty acids were purchased as their sodium salts from Sigma Aldrich chemical company (St. Louis, MO). Recombinant human TNF-α was purchased from Peprotech (Rocky Hill, NJ) and stock solutions were prepared in de-ionized water as per manufacturer’s instruction.

# 2.2 Cell Culture

For cytotoxicity measurements and western blots, HepG2/C3A cells were grown in 6-well plates, and treated with 2 ml of the control or FFA-containing medium, which contained 0.7 mM of palmitate, oleate or linoleate. Medium containing 4% (w/v) BSA was used as control. TNF-α was added from a 100 μg/ml stock in deionized water to make the desired final concentrations of either 20 or 100 ng/ml.

*Treatment notations:* The notation for the different treatment condition follows this scheme: Control medium (without any FFA or BSA) is denoted as ‘Control’ or ‘H’, medium containing BSA is denoted as ‘B’, palmitate-containing medium is denoted as ‘P’, oleate-containing medium is denoted as ‘O’ and linoleate-containing medium as ‘L’. The numbers following the medium name represent the concentration of TNF-α in ng/ml, e.g., the culture medium containing 0.7mM palmitate without TNF-α is designated Palm0 (or P-0); 0.7mM palmitate with 20 ng/ml TNF-α is named Palm20 (or P-20); 0.7 mM palmitate with 100 ng/ml TNF-α is named Palm100 (or P-100).

## 2.3 Measurement of cytotoxicity

The cytotoxicity of the treatments was measured as the fraction of lactate dehydrogenase (LDH) released into the medium as explained previously (Srivastava and Chan, 2007), using the cytotoxicity detection kit (Roche Applied Science, Indianapolis, IN).

**2.4 Measurement of ketone body release**

Acetoacetate and beta-hydroxybutyrate are two of the most important ketone bodies produced by liver cells. Therefore, ketone body release was measured as sum of acetoacetate and beta-hydroxybutyrate released into the medium. Acetoacetate release was measured fluorimetrically by an enzymatic assay based on consumption of NADH by the enzyme beta-hydroxybutyrate dehydrogenase in presence of acetoacetate (Mellanby and Williamson, 1974) and beta-hydroxybutyrate was measured by a commercially available assay (Stanbio Laboratories, Boerne, TX) according to manufacturer’s protocol.

**2.5 Microarray analyses**

Confluent cells on 10 cm tissue culture plates were exposed to different treatments for 24h. RNA was isolated with Trizol reagent. The expression profiles of about 19500 genes were obtained with cDNA microarrays, conducted according to protocols available online (http://www.vai.org/Research/Services/LMT/Protocols.aspx). Two biological and two technical replicates were employed for each condition. The microarray data has been deposited at the GEO website, with a query number of GSE5441.

Supplementary Figure S1 legends

**Supplemental Figure**

Supplemental Figure S1 (A) Variation of the discriminating ability of the mixture model with the smoothing constant (λ) and the weight to GO (τ). (B) Effect of randomly varying the values of the similarity matrix elements by 20% on the separation profile. (C) Separation profile with two of the non-toxic (H-0 and H-20) conditions removed. (D) Separation profile with non-toxic fatty acid (L-0 and L-20) conditions removed.
